# Retromammary fat, axillary and arm metastases from a retroperitoneal leiomyosarcoma: report of a case with an indolent behaviour

**DOI:** 10.3332/ecancer.2017.778

**Published:** 2017-10-31

**Authors:** Walberto Monteiro Neiva Eulálio Filho, Samuel Madeira Campos Melo, Rafaela de Brito Alves, Luiz Ayrton Santos Junior, Danilo da Fonseca Reis Silva

**Affiliations:** 1 Departamento de Oncologia—Oncomédica, Rua Paissandu, 1862, Centro, Teresina, PI, Brazil; 2 Universidade Federal do Piauí—UFPI Campus Universitário Ministro Petrônio Portella, Bairro Ininga, Teresina, PI, Brazil

**Keywords:** leiomyosarcoma, neoplasm metastasis, subcutaneous tissue

## Abstract

Leiomyosarcomas are sarcomas that originate within smooth muscle cells and generally occur in older patients. These tumours account for 10% of all soft-tissue sarcomas. Metastases occur most commonly to the lungs, kidneys, and liver. Cutaneous metastases may also occur but are usually a very rare and late event. We present a case of a 46-year-old woman who developed subcutaneous metastasis to the axilla, arm and breast after surgical resection of the primary tumour.

The patient maintained controlled disease with surgical resections and clinical follow-up, initiating chemotherapy one year after the diagnosis of metastatic disease.

## Background

Soft-tissue sarcomas are a heterogeneous group of malignancies with different morphological patterns of mesenchymal lineage, representing about 1% of malignancies in adults [1]. Leiomyosarcomas are sarcomas derived from the smooth muscle. These tumours generally occur in older patients and account for 10% of all soft-tissue sarcomas [4]. Primary leiomyosarcoma is most commonly found in the gynaecological, gastrointestinal and somatic soft tissue (retroperitoneum, in particular). The tumour may also arise from the skin and subdermis. In general, haematogenous dissemination occurs most commonly to the lungs, kidneys, and liver. Cutaneous metastasis, although very rare, may also occur. It is usually a late event and heralds a poor prognosis [3]. We present a case of a 46-year-old woman who developed subcutaneous metastases to the axilla, arm, and breast after surgical resection of the primary tumour.

## Case report

**Figure 1.**
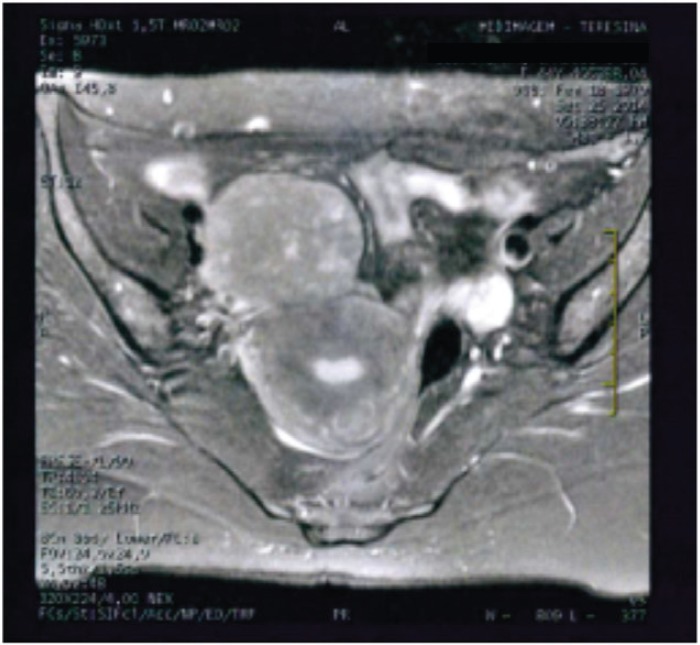
Axial T2-weighted MRI image showing a solid expansile lesion.

A 46-year-old female patient sought medical attention, complaining of lumbar pain and discreet abdominal discomfort. She underwent magnetic resonance imaging (MRI), which identified a solid mass of approximately 5 cm, located in the right paramedian region of the pelvic cavity (Figure 1). She was managed by a primary care physician who opted for a short interval follow-up. After three months, growth of the mass occurred and the patient was referred to oncology for surgical resection in November 2014. Histopathology revealed an intermediate-grade leiomyosarcoma, measuring 7.4 × 5.5 × 4.5 cm, confirmed by immunohistochemistry. Systemic staging of the patient was performed with pelvic MRI and computed tomography (CT) scan of the chest and abdomen, revealing no evidence of disease. The advantages and disadvantages were discussed with the patient and adjuvant radiotherapy was chosen (28-day cycle, at a dose of 56 Gy). Radiotherapy ended in April 2015. Follow-up care on a regular three-month basis with history-taking and physical examination was provided by the patient’s oncologist.

**Figure 2.**
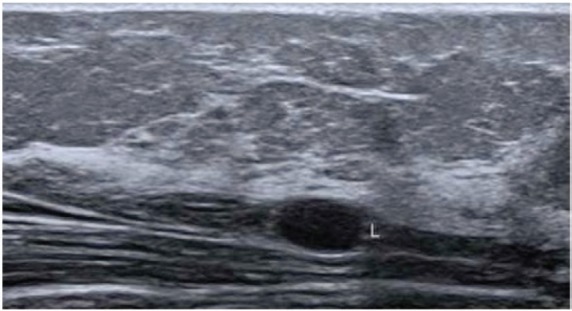
Ultrasonography showing subcutaneous fluid collection in the anterior aspect of the left arm root.

**Figure 3.**
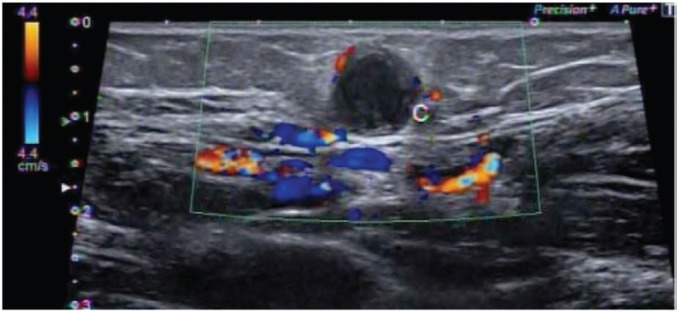
Ovoid hypoechoic nodule, located in the lateral quadrant of the right breast. Category BI—RADS 3.

**Figure 4.**
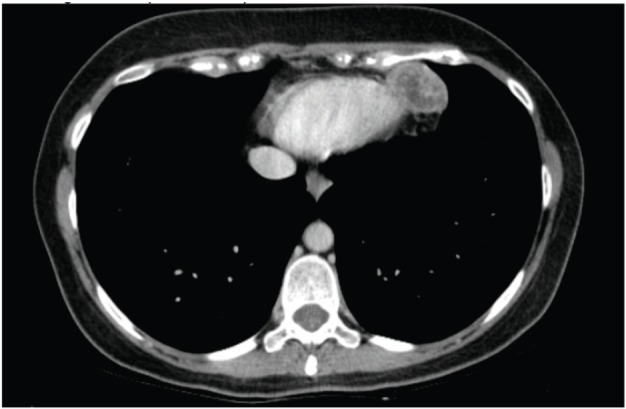
Computed axial tomography scan view showing secondary haematogenous implant in the pericardial fat.

**Figure 5.**
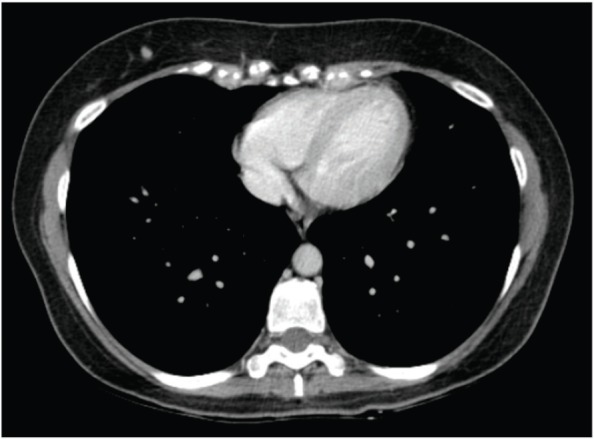
Computed axial tomography scan view showing secondary haematogenous implant in the right subcutaneous tissue of the breast.

**Figure 6.**
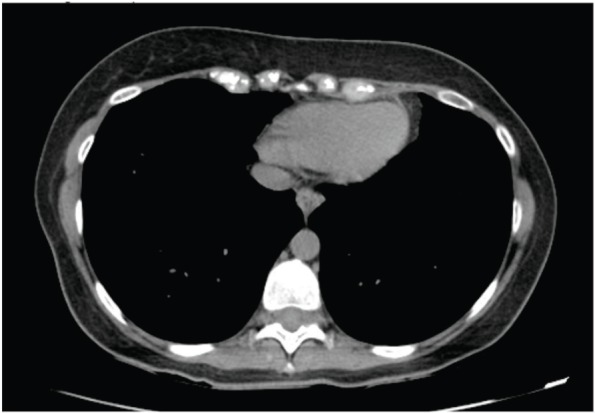
Computed axial tomography analysis after resection of secondary haematogenic implant.

In December, she noticed a round mobile palpable mass in the left axilla, measuring 2–3 cm. Ultrasound features were consistent with sebaceous cyst (Figure 2). In January 2016, she underwent tumour resection. Immunohistochemical study revealed the expression of desmin and smooth muscle actin in neoplastic cells, which was compatible with metastatic leiomyosarcoma. In April 2016, routine mammography associated with breast ultrasonography showed an ovoid hypoechoic nodule, with absence of flow on Doppler image, located in the retromammary/subaponeurotic fat plane, in the upper lateral quadrant of the right breast, category BI—RADS 3 (Figure 3). During the same period, a painful, mobile, palpable mass with ill-defined borders was noticed in the left arm. A new surgical resection of both masses was performed in May 2016 with histopathology confirmation of metastatic leiomyosarcoma. The patient underwent 18F-FDG PET/CT in June 2016 showing no metabolic changes or any suspected lesions. In January 2017, eight months after the last surgery, an asymptomatic mass was found in the pericardial fat (Figure 4) and another mass appeared in the right subcutaneous tissue of the breast (Figure 5). Since there was a risk of growth of the mass in pericardial fat tissue with compression of the heart, first-line chemotherapy was initiated and doxorubicin was scheduled to be administered every three weeks. After three cycles of chemotherapy, restaging CTs revealed that both masses had grown slightly. Thus, a videothoracoscopy was performed in May 2017 for the resection of the mass in pericardial fat tissue. The breast tumour was removed at the same time. The patient maintains a good quality of life and is currently disease free (Figure 6).

## Discussion

Leiomyosarcoma is a rare malignant tumour that arises from smooth muscle cells. Although leiomyosarcoma is well known for its metastatic potential, cutaneous metastasis is a remarkably uncommon event [4]. The majority of patients with cutaneous metastatic deposits from leiomyosarcomas also have concomitant metastatic deposits in other internal organs, indicating advanced disease and a poor prognosis. Cutaneous metastasis occurs in only 0.7–9% of all malignancies. Metastatic skin deposits from sarcomas are even rarer, accounting for 1–2.6% of all metastatic skin tumours. Of all sarcomas, leiomyosarcomas are more likely to originate cutaneous metastatic deposits [5]. The scalp seems to be the most common site for cutaneous metastasis in leiomyosarcomas, occurring in one-half of all patients with skin metastasis. This trend is probably explained by the abundant vascularity of the scalp and a tendency of leiomyosarcoma to disseminate via the haematogenous route [4]. Since these tumours spread primarily through the bloodstream, lymph node involvement is rare [6].

Retroperitoneal and intra-abdominal leiomyosarcoma, the most common subgroup, is associated with an aggressive clinical course. In contrast, leiomyosarcoma of the somatic soft tissues represent the second subgroup and has a better prognosis. Primary cutaneous leiomyosarcoma comprises the third subgroup and arises from the piloerector muscle of the dermis. These lesions present as pyogenic granuloma, basal cell carcinoma, squamous cell carcinoma, fibroids and node injury. Multiple lesions of cutaneous leiomyosarcoma should raise the possibility of abdominal or retroperitoneal metastatic disease [7].

Leiomyosarcomas grow slowly and may remain asymptomatic for a long period of time, delaying diagnosis and resulting in a worse prognosis [8]. Diagnosis should be made only after immunohistochemistry study, since conventional methods such as hematoxylin/eosin may fail to differentiate leiomyosarcoma from malignant fibrohistiocytomas [9].

A study involving 139 patients found that complete surgical resection without microscopic residual tumour and contamination is likely to offer the best chance of long-term survival. Aggressive surgery for recurrent sarcoma is recommended [10].

There have been few treatment options for advanced leiomyosarcomas. Chemotherapy is administered with palliative intent. It is usually associated with low response rates and may cause significant distress. However, it is still the cornerstone of treatment for metastatic soft-tissue sarcomas, reducing symptoms, decreasing tumour bulk and even prolonging survival. For patients with unresectable extrauterine leiomyosarcomas, metastatic disease is often treated with doxorubicin or doxorubicin-based combinations. Gemcitabine and docetaxel are often considered first-line or second-line treatment for leiomyosarcomas and are particularly effective in managing uterine leiomyosarcoma [2].

Surgical resection of metastatic disease can provide long-term relapse-free survival and probable cure in selected patients, the majority of whom have isolated pulmonary metastatic disease [11]. The patient described in this case report had superficial metastases and surgical treatment was planned. Although surgery was not curative, it was important to prolong the period free from palliative chemotherapy. Despite significant side-effects of chemotherapy, including reversible myelosuppression, mucositis, alopecia, nausea and vomiting, acute and chronic cardiotoxicity, need for regular medical check-ups and frequent blood sampling, a delay in starting chemotherapy is likely to be associated with a better quality of life. In the current case, disease control was achieved by surgical resections and clinical follow-up. The patient initiated palliative chemotherapy more than a year after the first metastasectomy.

## Conclusions

Clinicians should be aware of this unusual presentation. This case also draws attention to the importance of regular follow-up, including history taking and the performance of a meticulous physical examination in patients with leiomyosarcoma, to rule out subcutaneous metastasis. Furthermore, although palliative chemotherapy is standard practice for metastatic disease, this case highlights that metastasectomy can provide long-term relapse-free survival, postpone chemotherapy and may improve the quality of life in these patients. Hence, oligometastatic patients should be preferentially managed with surgical resection in leiomyosarcoma exhibiting indolent behaviour.

## Conflicts of interest

The authors have no conflicts of interest to declare.
